# Detection of coronary artery calcification with nontriggered computed tomography of the chest

**DOI:** 10.1590/0100-3984.2016.0181

**Published:** 2018

**Authors:** Gustavo Lemos Pelandré, Nathália Martins Pereira Sanches, Marcelo Souto Nacif, Edson Marchiori

**Affiliations:** 1 MD, Radiologist, Assistant Professor in the Department of Internal Medicine of the Universidade Federal de Santa Catarina (UFSC), Florianópolis, SC, Brazil; 2 Medical Student at the Universidade Federal de Santa Catarina (UFSC), Florianópolis, SC, Brazil; 3 Adjunct Professor in the Department of Radiology, School of Medicine, Universidade Federal Fluminense (UFF), Niterói, RJ, Brazil; 4 Full Professor at the Universidade Federal do Rio de Janeiro (UFRJ), Rio de Janeiro, RJ, Brazil

**Keywords:** Tomography, X-ray computed, Cardiovascular diseases, Coronary disease

## Abstract

**Objective:**

To evaluate the accuracy of visual analysis and of the coronary artery calcium (CAC) score in nontriggered computed tomography (CT), in comparison with that of the CAC score in electrocardiogram-triggered CT, in identifying coronary calcification.

**Materials and Methods:**

A total of 174 patients for whom CT was indicated for CAC scoring underwent nontriggered and triggered CT in a 64-channel multislice scanner, in a single session without a change in position. The images were interpreted by a radiologist with seven years of experience in thoracic and cardiovascular radiology. The measurement of coronary calcium was carried out by three methods: CAC score with dedicated software in nontriggered CT, CAC score with dedicated software in triggered CT, and visual analysis without dedicated software in nontriggered CT.

**Results:**

In nontriggered CT, the CAC score presented an accuracy of 95.98% (95% CI: 91.93-98.04). The visual analysis showed an accuracy of 97.13% (95% CI: 93.45-98.77).

**Conclusion:**

Nontriggered CT showed excellent accuracy in the identification and exclusion of coronary calcification, either the CAC score was determined with dedicated software or through visual analysis.

## INTRODUCTION

Cardiovascular disease is currently the leading cause of death in Brazil and worldwide. According to the World Health Organization, cardiovascular disease was responsible for 25% of all premature deaths worldwide in 2012, ischemic heart disease accounting for 13% and stroke accounting for 12%^(^^1^^)^. In Brazil, cardiovascular disease was reportedly responsible for 31% of all deaths in 2012^(^^2^^)^. Although studies on the economic impact of cardiovascular disease in Brazil are scarce, the costs attributable to such diseases in the country in 2004 were estimated to have exceeded 11 billion Brazilian reals, corresponding to 1.74% of the gross domestic product^(^^3^^)^.

A reduction in the incidence of cardiovascular disease, which would lead to a reduction in the associated morbidity and mortality, has been the objective of public health policies in Brazil and in other countries. There is a growing interest in primary prevention, and it is therefore essential to identify individuals at high risk of developing cardiovascular disease, in order to define individual therapeutic goals more accurately^(^^4^^)^.

The risk of cardiovascular disease is estimated by calculating the sum of the risk of each of the risk factors plus the added risk resulting from synergism among some of those factors. Given the complexity of these interactions, various algorithms, based on regression analyses of population studies, have been created. The Fifth Brazilian Guidelines on Dyslipidemias and Prevention of Atherosclerosis recommend the use of an *overall risk score* for 10-year risk assessment and an *age-adjusted risk score* as an option for individuals over 45 years of age who are considered to be at low or intermediate risk in the 10-year risk assessment. Those scores are based on the following variables: gender; age; cholesterol levels; blood pressure; history of smoking; and the presence or absence of diabetes. From the scores obtained, the individual 10-year risk of presenting one the main cardiovascular events can be estimated^(^^4^^)^ : coronary artery disease, stroke, peripheral arterial occlusive disease, or heart failure.

Although quite useful, clinical scores in isolation have a limited capacity for stratifying cardiovascular risk. Some clinical and imaging tests can play important roles as complements to clinical scores in the stratification of risk in asymptomatic patients. For individuals deemed to be at intermediate risk on the basis of the clinical scores, certain aggravating factors can reclassify the risk as high and should therefore be taken into consideration^(^^4^^)^.

With the advent of multi-detector computed tomography (MDCT), it is now possible to detect coronary calcification in a noninvasive manner and with excellent accuracy. The recommended technique is the determination of the coronary artery calcium (CAC) score, using electrocardiogram-triggered CT (hereafter, triggered CT) and dedicated software based on the Agatston method^(^^5^^)^. The use of the CAC score allows the stratification, discrimination, and reclassification of the cardiovascular risk established on the basis of clinical criteria^(^^6^^)^.

Although determination of the CAC score is a noninvasive, highly accurate means of assessing coronary calcification, it is not widely available in Brazil, because it requires the use of cardiac catheters, a scanner with a high number of detectors, and specific software. Studies employing nontriggered CT have shown that visual (non-quantitative) identification of coronary calcification provides relevant clinical information^(^^7,8^^)^ and presents a positive statistical correlation with increased cardiovascular mortality^(^^9,10^^)^.

The objective of this study was to evaluate visual analysis and the CAC score, both determined by nontriggered CT, in comparison the CAC score determined by triggered CT, in terms of their accuracy in the identification or exclusion of coronary calcification.

## MATERIALS AND METHODS

This was a prospective, descriptive, observational, analytical study, approved by the Research Ethics Committee of the Cancer Research Center of the Federal University of Santa Catarina, in the city of Florianópolis, Brazil. We included patients for whom a CT scan was indicated in order to determine the CAC score, between October 1, 2014 and July 31, 2015. Patients with a history of surgical manipulation of the coronary arteries (angioplasty or myocardial revascularization) were excluded. All participating patients gave written informed consent.

All patients underwent two CT examinations in a 64-channel MDCT scanner, in a single session, with no change in position, at the lowest radiation doses possible. The first examination was non-contrast-enhanced, volumetric acquisition, nontriggered CT of the chest, with coverage from the apex to the base of the lung, performed during a single breath-hold, with 1.25 mm-thick slices. The second examination was a triggered cardiac CT scan, with a series of 3 mm-thick axial slices covering the entire extent of the heart.

The images were interpreted by a radiologist with seven years of experience in thoracic and cardiovascular radiology. The calcification of the coronary arteries was measured by three different methods: CAC score determined (by the Agatston method) in triggered CT; CAC score determined (by the Agatston method) in nontriggered CT; and visual analysis in nontriggered CT. The images were interpreted at different times for each method.

In the images obtained by triggered and nontriggered CT, we used dedicated software, based on the Agatston method, for the quantification of the CAC score. In the Agatston method, calcification is defined as a hyperattenuating lesion with a density > 130 Hounsfield units (HU) and area > 3 adjacent pixels (≥ 1 mm^3^ ). The software identifies the calcified lesions and calculates the CAC score for each by multiplying the area in pixels by the maximum density score (1 for 130-199 UH; 2 for 200-299 UH; 3 for 300-399 UH; and 4 for ≥ 400 UH), after which it totals the scores of the lesions. The CAC scores were categorized as follows^(^^11^^)^ : 0 = no calcification; 1-10 = minimal calcification; 11-100 = mild calcification; 101-400 = moderate calcification; 401-1000 = severe calcification; and > 1000 = extremely severe calcification.

**Figure 1:**
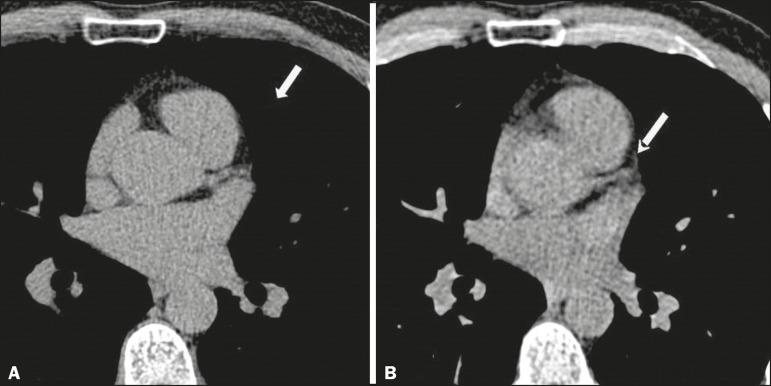
Triggered CT (**A**) and nontriggered CT (**B**), showing the absence of calcification in the trunk of the left coronary artery (arrow).

**Figure 2:**
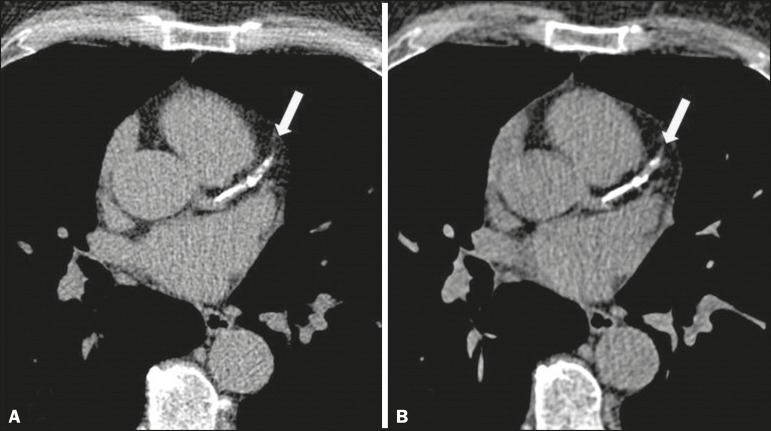
Triggered CT (**A**) and nontriggered CT (**B**), showing extensive calcification in the proximal anterior descending coronary artery (arrow).

We performed the visual analysis with the nontriggered CT images, using a high-resolution (3000 dpi) monitor for the interpretation of the examinations and a mediastinal window (level = 30-60 UH; width = 300-500 UH), based on axial reconstructions. The arterial branches were classified, in terms of the presence or absence of calcification, through subjective analysis without the use of specific software, in a manner similar to that employed by other authors^(^^11^^)^. No measurements or quantifications were performed during the visual analysis, which was used only to identify coronary calcification (Figures 1 and 2).

Positive predictive value (PPV), negative predictive value (NPV), sensitivity, specificity, and accuracy, with their respective 95% confidence intervals (95% CIs), were calculated using the OpenEpi program. The CAC scores were expressed in numerical values, organized by category.

## RESULTS

We evaluated 174 patients, of whom 103 were male (59.2%) and 71 were female (40.8%). Patient ages ranged from 24 to 90 years (mean, 58.4 years). On the triggered CT scans, a CAC score of zero was observed in 69 (39.6%) of the 174 patients evaluated, compared with 72 (41.37%) on the nontriggered CT scans. The CAC scores obtained from the triggered CT scans were similar to those obtained from the nontriggered CT scans (Table 1).

**Table 1:** Distribution of the patients by CAC score (Agatston method) determined by triggered and nontriggered CT.

	Measures of CAC score	
Category	Triggered CT	Nontriggered CT
0	69	72
< 10	26	20
< 100	37	34
< 400	24	28
< 1000	11	12
≥ 1000	7	8

For the detection or exclusion of coronary calcification, the accuracy of the CAC score determined by nontriggered CT (in comparison with that determined by triggered CT) was 95.98% (95% CI: 91.93-98.04), with a specificity of 97.1 (95% CI: 90.93-99.2), a sensitivity of 95.24% (95% CI: 89.33-97.95), an NPV of 93.06% (95% CI: 84.75-97.0), and a PPV of 98.04% (95% CI: 93.13-99.46). For the detection or exclusion of coronary calcification, the accuracy of the visual analysis of nontriggered CT images (in comparison with that of triggered CT images) was 97.13% (95% CI: 93.45-98.77), with a specificity of 98.55 (95% CI: 92.24-99.74), a sensitivity of 96.19% (95% CI: 90.61-98.51), an NPV of 94.44% (95% CI: 86.57-97.82), and a PPV of 99.02% (95% CI: 94.65-99.83).

The false-negative results obtained from the nontriggered CT scans (CAC scores and visual analysis scores) were identified by near-zero CAC scores obtained from the triggered CT scans (Table 2). Among the nontriggered CT findings for the presence of coronary calcification, false-negative results were obtained in only five patients (6.94%), with a mean CAC score of 1.6 (maximum, 2.9), and false-positive results were obtained in only two (1.96%), with a mean CAC score of 0.75 (maximum, 1.1). Among the visual analysis findings for the presence of coronary calcification, false-negative results were obtained in only four patients (5.56%), with a mean CAC score of 1.92 (maximum, 2.9), and a false-positive result were obtained in only one (0.98%).

**Table 2:** Distribution of the CAC scores determined by triggered CT, stratified by CAC score of zero in nontriggered CT and in the visual analysis.

	CAC score of zero on nontriggered CT				
	Agatston method			Visual analysis	
Category	N	%		N	%
0	67	93.06		68	94.44
< 10	5	6.94		4	5.56
< 100	-	-		-	-
< 400	-	-		-	-
< 1000	-	-		-	-
≥ 1000	-	-		-	-

## DISCUSSION

Imaging studies of the cardiovascular system have been the subject of recent publications in the radiology literature of Brazil^(^^11-16^^)^. Some studies have proposed that dedicated cardiac CT for the detection/quantification of coronary calcium is not necessary in patients for whom CT of the chest is negative^(^^17^^)^. In the present study, the CAC score by nontriggered CT and the visual analysis by nontriggered CT both showed high accuracy (95.98% and 97.13%, respectively, compared with the CAC score by triggered CT) for the detection or exclusion of coronary calcium.

Comparing the CAC score by triggered CT and nontriggered CT, Wu et al.^(^^18^^)^ analyzed 483 patients and found that the CAC score by nontriggered CT showed a PPV of 97-98% and an NPV of 98-99%. The authors also found that the CAC score by nontriggered CT was miscategorized in patients, most of whom were in a lower risk group based on the CAC score by triggered CT. In their study, there were five false-positive results and five false-negative results. For the false-negative nontriggered CT results, the mean CAC score by triggered CT was 6.2. For the falsepositive results, the mean CAC score by nontriggered CT was 4.5, which corresponds to a degree of calcification that is associated with low cardiovascular risk. All the miscategorized scores were 12 or less.

In a study of 55 patients diagnosed with chronic obstructive pulmonary disease, Budoff et al.^(^^19^^)^ compared the CAC score by triggered CT and nontriggered CT, reporting similar results. The authors found a CAC score of zero in 17 (34%) patients by triggered CT, as well as by nontriggered CT

In a meta-analysis, Xie et al.^(^^20^^)^ analyzed the correlation between the CAC score determined by triggered CT and that determined by nontriggered CT. Their systematic review included five studies, collectively involving 1316 asymptomatic participants. The CAC score by nontriggered CT was calculated in four of those studies, collectively involving 1153 participants, 625 of whom had a CAC score greater than zero on the triggered CT. Of those 625, 55 (8.8%) had a CAC score of zero on the nontriggered CT: 52 (8.3%) with a CAC score between 1 and 100 on triggered CT; and 3 (0.5%) with a CAC score between 100 and 400 on triggered CT.

Various authors have used semiquantitative techniques to evaluate coronary calcification on nontriggered CT scans. Einstein et al.^(^^21^^)^ found a strong association between the CAC score determined with a visual scale and that determined by the Agatston method, with high rates of agreement among the two for the six categories analyzed. Shemesh et al.^(^^9^^)^ categorized the calcification in each coronary artery segment as absent, mild, moderate, or severe (scores from 0 to 3, respectively), identifying a good correlation between cardiovascular mortality and a total score (for all segments combined) > 4. In a retrospective study, Jacobs et al.^(^^22^^)^ assessed the incidence of cardiovascular events in patients undergoing nontriggered CT. For evaluation of coronary calcification, those authors also assigned scores of 0 to 3 for each coronary artery segment. The authors found that the risk of a cardiovascular event increased in parallel with increases in the category of coronary calcification after adjustment for age, gender, clinical indication, image quality, and institution at which the examination was performed.

Comparing different methods for measuring coronary calcification on nontriggered CT scans, Chiles et al.^(^^10^^)^ found a good correlation between subjective visual assessment (absence of calcification, mild calcification, moderate calcification, or severe calcification) and increased risk of acute myocardial infarction and death. Among the patients studied by those authors, an absence of coronary calcification was found for 26.8% of those evaluated by subjective analysis and 27.7% of those evaluated by modified Agatston score.

Using nontriggered CT with visual analysis, Huang et al.^(^^23^^)^ analyzed 369 patients comparing the CAC score with that obtained by triggered CT and found false-negative results in 24 cases, the CAC score in those cases ranging from 1.1 to 21.1 (indicating mild calcification). Kirsch et al.^(^^24^^)^ evaluated the correlation between the CAC score by triggered CT and the visual analysis by nontriggered CT in 163 asymptomatic patients. The absence of coronary calcium on the visual analysis was associated with a CAC score on triggered CT ranging from 0 to 19 (indicating little or no calcification). Those values reflect the importance of nontriggered CT in the detection of coronary calcification, especially in contexts in which triggered CT is not available, nontriggered CT representing a more accessible alternative for evaluation of the coronary artery.

This study has certain limitations. The exams were performed at a single center in a single 64-channel scanner. The images were interpreted by one radiologist with experience in cardiovascular CT, and it is therefore not possible to estimate the effect of interobserver variations or accuracy in the visual identification of coronary calcium among less experienced professionals. However, studies comparing observers with different levels of experience did not identify significant interobserver variations in the visual analysis of coronary calcification^(^^10,18,21^^)^.

## CONCLUSION

Nontriggered CT showed excellent accuracy in the identification and exclusion of coronary calcification when compared with triggered CT, either by the CAC score with dedicated software or by visual analysis. Among the non triggered CT results, there were no significant differences between the CAC score and the visual analysis, in terms of accuracy.
